# Whole-Genome Sequencing of a Brucella melitensis Strain (BMWS93) Isolated from a Bank Clerk and Exhibiting Complete Resistance to Rifampin

**DOI:** 10.1128/MRA.01645-18

**Published:** 2019-08-15

**Authors:** Zhi-guo Liu, Xiao-an Cao, Miao Wang, Dong-ri Piao, Hong-yan Zhao, Bu-yun Cui, Hai Jiang, Zhen-jun Li

**Affiliations:** a State Key Laboratory for Infectious Disease Control and Prevention, National Institute for Communicable Disease Control and Prevention, Chinese Center for Disease Control and Prevention, Beijing, China; b Inner Mongolia Autonomous Region Comprehensive Center for Disease Control and Prevention, Huhhot, China; c Ulanqab Center for Endemic Disease Control and Prevention, Jining, China; d State Key Laboratory of Veterinary Etiological Biology, Lanzhou Veterinary Research Institute, Chinese Academy of Agricultural Sciences, Lanzhou, China; University of Arizona

## Abstract

Human brucellosis has become the most severe public health problem in the Ulanqab region of Inner Mongolia, China. Brucella melitensis BMWS93 was obtained from a blood sample taken from a bank clerk in the Ulanqab region of Inner Mongolia, China, and antimicrobial susceptibility testing *in vitro* showed no zone of inhibition, which confirmed resistance to rifampin. Therefore, whole-genome sequencing of this isolate was performed to better understand the mechanism of this resistance.

## ANNOUNCEMENT

Brucella melitensis is a Gram-negative facultative intracellular pathogen that causes abortion in goats and sheep and Malta fever in humans (1). The disease causes severe morbidity in humans and results in serious economic losses in livestock due to abortion and infertility (2). B. melitensis bv. 3 was the predominant biovar in Ulanqab (3). We added 5 ml blood from the bank clerk into brucella agar slope medium at 37°C under microaerobic conditions. BMWS93 was identified as B. melitensis bv. 3 and exhibited complete resistance to rifampin *in vitro* (4). Subsequently, *rpoB* gene sequencing demonstrated that *rpoB* gene mutations were not present in this isolate. Here, we report the whole-genome sequence of B. melitensis strain BMWS93 from the Ulanqab region of Inner Mongolia, China.

Genomic DNA of BMWS93 was extracted using a QIAamp DNA minikit, according to the manufacturer's instructions. After extraction, sequencing was conducted on a Pacific Biosciences RS II platform utilizing single-molecule real-time (SMRT) technology and a SMRTbell version 1.0 template prep kit for library preparation to determine the complete genomic sequence of B. melitensis BMWS93. The average read length was 6,625 bp, and the low-quality reads were filtered out using Trimmomatic version 0.38 (5). The filtered reads were assembled using SMRT Portal (6, 7) to generate scaffolds. The annotation was performed using GeneMarkS (8), RepeatMasker (9), Tandem Repeats Finder (TRF) (10), tRNAscan-SE (11), RNAmmer (12), and PHAST (13).

The genome size of BMWS93 is 3.30 Mb distributed over two circular chromosomes of 2.12 and 1.19 Mb, with 57.25% G+C content. Circular chromosomes 1 and 2 consist of 2,126,063 and 1,186,194 bases, respectively. The whole genome contains 3,321 coding genes. There are four types of repeat sequences and three kinds of tandem repeat sequences in the genome of this strain. The relative numbers of long terminal repeats, DNA transposons, long scattered repeat sequences, and short scattered repeat sequences are 15, 10, 4, and 8, respectively. There are 83 tandem repeat sequences, 66 minisatellite sequences, and 1 microsatellite sequence. There are 3 bacterial rRNA types (5S, 16S, and 23S), 2 small RNAs (sRNAs), and 55 tRNA operons. In addition, there were 11 genomic islands, 2 prophages, and 2 efflux transporter systems in the genome in this strain. Moreover, efflux transporter systems may contribute to this B. melitensis isolate’s resistance to rifampin (14). A comprehensive bioinformatics analysis could help to better understand the evolution, host specificity, and pathogenicity of B. melitensis.

### Data availability.

The whole-genome sequence of B. melitensis strain BMWS93 was deposited at DDBJ/EMBL/GenBank under the accession numbers CP034103 and CP034104 for chromosomes 1 and 2, respectively. The version described in this paper can be found under NCBI BioProject number PRJNA505925 and NCBI BioSample number SAMN10439570. The raw reads of sequenced genomic DNA of B. melitensis BMWS93 were deposited in the SRA under accession number SRR9311569.
